# Sexual and Reproductive Health Research and Research Capacity Strengthening in Africa: Perspectives from the region

**DOI:** 10.1186/s12978-015-0055-2

**Published:** 2015-07-31

**Authors:** Richard Adanu, Michael T. Mbizvo, Adama Baguiya, Vincent Adam, Beyene W. Ademe, Augustine Ankomah, Godwin N. Aja, Ademola J. Ajuwon, Olapeju A. Esimai, Taofeek Ibrahim, Dintle K. Mogobe, Özge Tunçalp, Venkatraman Chandra-Mouli, Marleen Temmerman

**Affiliations:** School of Public Health, College of Health Sciences, University of Ghana, Accra, Ghana; University of Zimbabwe, Avondale, Harare, Zimbabwe; Institut de recherche en science de la sante IRSS, Ouagadougou, Burkina-Faso; Department of Community Health College of Medical Sciences, University of Benin, Benin City, Nigeria; College of Public Health and Medical Sciences, Jimma University, Addis Ababa, Ethiopia; School of Public Health, Babcock University, Ilishan Remo, Nigeria; Department of Health Promotion and Education, Faculty of Public Health, College of Medicine, University of Ibadan, Ibadan, Nigeria; Department of Community Health, Obafemi Awolowo University, Ile-Ife, Nigeria; Department of Community Health, Usmanu Danfodiyo University & Teaching Hospital, Sokoto, Nigeria; Faculty of Health Sciences, University of Botswana, Gaborone, Botswana; Department of Reproductive Health and Research including UNDP/UNFPA/UNICEF/WHO/World Bank Special Programme of Research, Development and Research Training in Human Reproduction (HRP), World Health Organization, Geneva, Switzerland

**Keywords:** Sexual and reproductive health, Africa, Research, Policy, Capacity strengthening

## Abstract

Developing the capacity to effectively carry out public health research is an integral part of health systems at both the national and global levels and strengthening research capacity is recognized as an approach to better health and development in low- and middle-income countries (LMICs). Especially fields such as sexual and reproductive health (SRH) would require inter-disciplinary teams of researchers equipped with a range of methodologies to achieve this. In November 2013, as part of the International Family Planning Conference in Addis Ababa, Ethiopia, a group of African researchers came together to discuss the gaps and strategies to improve sexual and reproductive health research and research capacity strengthening in Africa. This commentary summarizes the three broad areas where the issues and proposed solutions have concentrated around:

1. Addressing research gaps that are most relevant to policies and programmes in SRH,
2. Carrying out high quality and collaborative research, and
3. Translating research findings into SRH policies and programmes.

Even though the focus of the discussions was Africa, the issues and proposed solutions can also be applied to other regions facing a high burden of disease with limited resources. The time is now and these can be achieved through synergistic commitment of African and global researchers, funders and organizations.

## Background

Developing the capacity to effectively carry out public health research is an integral part of health systems at both the national and global levels and strengthening research capacity is recognized as an approach to better health and development in low- and middle-income countries (LMICs).[1] The process of embedding research within health systems requires competent, locally-based scientists and a strong supportive and enabling environment that will allow research communities to grow and deliver research that contribute to the health of their communities.[2] Especially fields such as sexual and reproductive health (SRH) would require inter-disciplinary teams of researchers equipped with a range of methodologies to achieve this.

 The UNDP/UNFPA/UNICEF/WHO/World Bank Special Programme of Research, Development and Research Training in Human Reproduction (HRP) has been leading large multi-country research studies as well as research capacity strengthening efforts in low- and middle-income countries (LMICs) since its inception more than 40 years ago.[3] In November 2013, as part of the International Family Planning Conference in Addis Ababa, Ethiopia, a group of African researchers came together to discuss the gaps and strategies to improve SRH research and research capacity strengthening in Africa. The day-long discussions and proposed solutions have focused around the following three broad areas: Addressing research gaps that are most relevant to policies and programmes, carrying out high quality and collaborative research, and translating research findings into policies and programmes. Even though the focus of the discussions was Africa, the issues and proposed solutions can also be applied to other regions with limited resources facing a high burden of disease.

### Addressing research gaps that are most relevant to policies and programmes in SRH

Currently, in practice, international organizations and networks tend to decide on SRH research priorities for Africa, based on their areas of focus and mandate. Some of the research questions they identify are extremely relevant, others are less so. Sometimes, African policy makers, programme managers (within the government and non-government sectors) and researchers are closely involved in defining research priorities. More often, their participation is token or non-existent.

To address this problem, funders and government officials should identify SRH priorities at the continental and national levels through a credible and consultative process. This will ensure that external funding as well as the funds from African governments and non-governmental organizations are used to address research gaps that are most relevant to continental, regional and country-specific SRH policies and programmes. Moreover, they can insist on the central role of African policy makers, programme managers, health workers and researchers in setting these priorities.

### Carrying out high quality and collaborative SRH research

Another problem identified related to the fact that African research institutions and African researchers often play an ancillary role in major SRH research projects carried out in the continent. Much of the research is led by research institutions and researchers from high-income countries. Therefore, empowering existing researchers and institutions, while building the next generation of African researchers will be paramount.

(i) Empowering existing African researchers and research institutions and drawing from their expertise

There are a number of actions which can be taken by different stakeholders. Staff in research institutions should receive refresher training in areas of emerging interest, for example implementation research and costing studies are two major areas of focus in which few African research institutions have expertise. Meanwhile, funders and government officials should insist on the central role of African institutions and African researchers in research on SRH in the continent. Furthermore, they can promote mechanisms for information exchange - research institutions and researchers are not always aware of research that is under way in their countries or of new opportunities. County-level information sharing platforms could address this important gap. E-platforms could play a useful role here. Similarly information sharing could be valuable at the sub-continental and continental levels through organizations such as Association of Schools of Public Health in Africa.[4]

(ii) Building the next generation of African researchers in Africa:

Investment in training more researchers is crucial. There are some well-staffed and well-funded universities carrying out high quality research in Africa. These universities train researchers and provide them with opportunities to contribute to and learn from research projects that are under way. But such universities are few and far between. To quicken the pace and expand the number of trained researchers across the continent, more training and more learning-by-doing opportunities must be created. An integral part of the institutional support must be directed at supporting senior staff to devote time and effort to identify, train and mentor promising young researchers. This support must also be coupled by creating funding opportunities for young researchers such as earmarked funding created for junior researchers to compete for research funds.

### Translating research findings into SRH policies and programmes

The findings of many research studies conducted in Africa are presented in international meetings and published in international journals, but they are not communicated to the same extent (or received with the same interest) within the continent. Research institutions and researchers may think that working to take research findings to practice is not their responsibility; even if they do, they may not have the capacity nor the incentives to do this.

Therefore, it is important that research institutions include it in their mandate to train researchers in communicating the findings of their research to decision makers (policy makers, programme managers and funders), to civil society bodies and the population, as consumers of healthcare and target of any research, at large. They can also create incentives for researchers to reach out to stakeholders who could contribute to translating research findings into practice.

Institutional changes alone will not be sufficient. Researchers also will need to engage with the leaders and members of the communities where they carry out their research. They can use their privileged position as respected community members to share research findings with influential individuals and organizations to ensure that laws, policies and strategies are evidence-based, and research findings are effectively used to address communities’ sexual and reproductive health related priorities. Funders and government officials support this exchange by creating opportunities for researchers and programmers to get together periodically to share and discuss research findings as well as viewpoints and experiences.

## Conclusions

Availability of adequate funds is key to carrying out the actions listed above. Development and funding agencies supporting SRH work in Africa should find ways and means of using the available resources more effectively e.g. pooling resources to carry out a package of actions on setting research priorities, carrying out research and taking research findings to policy and practice, while building the capacity of research institutions and researchers in the process. In this respect, in 2014, HRP formed ‘the HRP Alliance’ to enhance regional research networks and mentoring capacity. HRP long-term institutional development grantee organizations, research partners and WHO Collaborating Centres comprise the HRP Alliance at regional levels. [3] Moreover, recent global initiatives such as Accelerating Excellence in Science in Africa (AESA) are very welcome and exciting developments.[5] The time is now and what we are proposing here can and will be achieved through synergistic commitment of African and global researchers, funders and organizations.

## Abbreviations

AESA: Accelerating excellence in science in Africa
RHR: Reproductive health and research
SRH: Sexual and reproductive health
HRP: The UNDP/UNFPA/UNICEF/WHO/World Bank Special Programme of Research, Development and Research Training in Human Reproduction
